# Predictors of sustainment of two distinct nutrition and physical activity programs in early care and education

**DOI:** 10.3389/frhs.2022.1010305

**Published:** 2022-11-09

**Authors:** Taren Swindle, Laura L. Bellows, Virginia Mitchell, Susan L. Johnson, Samjhana Shakya, Dong Zhang, James P. Selig, Leanne Whiteside-Mansell, Geoffrey M. Curran

**Affiliations:** ^1^Department of Family and Preventive Medicine, College of Medicine, University of Arkansas for Medical Sciences, Little Rock, AR, United States; ^2^Division of Nutritional Sciences, Cornell University, Ithaca, NY, United States; ^3^Independent Researcher, Little Rock, AR, United States; ^4^Section of Nutrition, Department of Pediatrics, University of Colorado Anschutz, Aurora, CO, United States; ^5^Department of Biostatistics, College of Public Health, University of Arkansas for Medical Sciences, Little Rock, AR, United States; ^6^Department of Pharmacy Practice, College of Pharmacy, University of Arkansas for Medical Sciences, Little Rock, AR, United States; ^7^Center for Mental Health and Outcomes Research, Central Arkansas Veterans Heathcare System, Little Rock, AR, United States

**Keywords:** implementation research, sustainability, early care and education, childcare, nutrition, physical activity, implementation science

## Abstract

**Introduction:**

The goal of the present study was to investigate factors associated with sustainment of two evidence-based programs for nutrition promotion in early care and education (ECE) settings – Food Friends (FF) and Together, We Inspire Smart Eating (WISE).

**Materials and methods:**

In a cross-sectional study design, ECE directors (*N* = 55) from centers that had previously been trained in WISE or FF completed a survey. Program-specific measures included Steckler's Perception of Innovations, the Program Sustainability Assessment Tool (PSAT), and the Organizational Readiness for Change Assessment (ORCA). For our primary outcomes, two measures of sustainment were examined: Nutrition Continued Practice (i.e., the use of or general focus on nutrition programs) and Program Fidelity (i.e., how well centers used specific evidence-based practices of WISE or FF). Multiple regression was used to determine the association of these outcomes with program, years since last implementation, and overall scores on predictors. Follow-up correlation analyses were used to investigate outcome relationships with context submeasures due to high intercorrelations between predictor submeasures.

**Results:**

Nutrition Continued Practice was significantly predicted by program and overall PSAT score. WISE programs had significantly higher Nutrition Continued Practice scores than FF program (*p* = 0.03). All subscales of the PSAT (e.g., environmental support, funding stability, organizational capacity, program adaptation, communications, and strategic planning) were significantly correlated with Nutrition Continued Practice (all rs > 0.30, all ps < 0.03). Program Fidelity was significantly predicted by PSAT and Steckler Perception of Innovation scores. All subscales of the PSAT were strongly positively correlated with Program Fidelity (all rs > 0.48, all ps < 0.001); relative advantage (r = 0.54, *p* < 0.001) and level of institutionalization (r = 0.61, *p* < 0.001) were positively correlated with Program Fidelity.

**Conclusion:**

This study suggests that factors associated with the continued practice of program principles are partially distinct from those that are associated with the sustainment of specific practices driving program fidelity. Results suggest capacity building strategies may be important for both continued attention to nutrition and physical activity as well as sustaining fidelity to specific evidence-based practices.

## Introduction

Healthy eating (1–3), regular physical activity (3–5), and maintaining a healthy body weight (3, 6, 7) are established preventive measures to curb risk for a range of diseases including cardiovascular diseases, non-alcoholic liver diseases, metabolic syndrome, diabetes, and several cancers. However, most children do not meet recommendations for healthy diet and physical activity (PA) (8–14). Establishing early nutrition and PA habits are important for lifelong health and healthy weight (3, 15). Early care and education (ECE) environments are promising settings for promoting nutrition and PA for children. In the United States (U.S.), 12.5 million of children under 5 years spend approximately 30 hours in ECE centers per week (16, 17). In other high-income countries, usage rates are similarly high; 45% of children under 5 years of age in Australia are in childcare (18), and over 80% of children in the European Union receive formal childcare before attending compulsory school (19). Establishing and sustaining effective programs in ECE settings may have a significant, positive effect on child health.

Sustainability is the endurance of a program after a defined program period and after the ending of external implementation support, which is characterized by (a) the integration of the program in an existing institutional or community system (20, 21) (b) the continuation of the intervention (14, 20), and (c) progress in target behavior, yielding continued gains to the target population (20). Sustaining programs for promoting child health has proved more challenging than establishing initial implementation of such programs (22, 23). Specifically, there have been many public health efforts implemented to prevent and control childhood obesity, but lack of sustainment of program/intervention efforts is a major translational issue in public health (23–25). In fact, 40 to 60% of interventions are not sustained after external funding ends (22, 25–29). Implementation science recognizes that closing such gaps in sustainment of programs is crucial to achieve continued benefits for the target population (20, 30) and to maintain community engagement (25, 30).

Reflecting the growing emphasis on sustainability in implementation, there are several theories, models, and frameworks dedicated to understanding this topic (31). One of the most prominent models, the Dynamic Sustainability Framework (DSF), posits that characteristics that influence program sustainment include internal context (e.g., staff availability, program budget), external context (e.g., political support for a program or for the needs a program serves), and program-specific components (e.g., how fun or engaging a program is perceived to be), and the interaction among these (32). Recent systematic reviews (23, 25, 33), although not informed by the DSF in their framing and design, have supported the framework by identifying factors that align with key DSF constructs for predicting sustainment in educational settings. Internal contextual barriers to sustainment included lack of staff and staff turnover, time, training, and general financial resources; external contextual barriers included community, political engagement, and parental involvement; program-specific barriers included teacher perceptions of how interesting or fun the program was and how adaptable the program was to individual center needs.

Across these reviews, only two studies were identified that examined sustainment of obesity prevention or nutrition promotion programs in ECE. Whether the general pattern of key factors for sustaining programs holds in the ECE setting is unknown. Ward and colleagues used a mixed methods approach to assess factors related to sustainment of the Healthy Start-Départ Santé intervention program after 2 years of the initial training in 140 ECEs in Canada (34). Qualitative interviews suggested lack of time, resistance among childcare staff, and low parental involvement as barriers while facilitators included support from policy to implement the program, budget-friendly menu, and staff engagement. In Illinois, U.S., Allar et al. (35) investigated the use of a physical activity program (I am Moving, I am Learning) approximately 10 years after initial implementation in Head Start, a government-funded program that serves children from families with low incomes. These authors identified that low equipment requirements, and the fun, flexible nature of the movement program were perceived as contributors to the sustainment of this program by teachers and parents (35). Additionally, the integration of this program into the regular classroom routine was also identified as being important for sustainment. Despite the importance of sustaining childhood obesity intervention programs and the potential for ECE as a target setting for sustaining such programs, there are limited studies that examine the sustainability of childhood obesity prevention programs in ECE. Investigating factors associated with sustainment in the context of ECE offers opportunity to test empirical theories such as the DSF.

The current study addresses this research gap by identifying barriers and facilitators of the sustainability of two intervention programs in ECEs in the United States: (1) Food Friends^®^ (FF), which includes Fun with New Foods and Get Movin' with Mighty Moves and (2) Together, We Inspire Smart Eating (WISE)^®^. Both programs have a focus on nutrition; FF also has a PA component. Specifically, the purpose of the present study was to understand sustainment factors associated with continued use of FF and WISE over time, as well as any factors that might be unique to the sustainment of each program. To that end, directors of centers that had or were currently implementing FF and WISE completed a survey that assessed (a) continued attention to nutrition and physical activity support at their center and (b) current FF and WISE fidelity, (c) internal and external contextual factors related to sustainment and program-specific components (e.g., how successful they perceived the program to be at their center, how the program compared to alternative options, how often the program was used at their center).

## Materials and methods

### Interventions

Food Friends is a preschool program implemented mainly in Colorado, U.S. that was designed to address healthful eating behaviors and PA patterns in preschoolers (i.e., children ages 3 to 5). FF includes offering new foods and taste tests over 18 weeks; teachers are trained to role model trying the new foods. There are 8 FFs mascots that introduce children to each food group. FF has a companion program, Mighty Moves, focused on supporting development of motor skills through structured activities, music, and classroom enhancements (e.g., scarves). It was implemented successfully for over 20 years and has been shown to both increase children's willingness to try and consume novel foods (food preference) and improve gross motor performance in the short-term (36, 37) and longitudinally (38, 39).

WISE was similarly designed to increase healthy eating habits in early childhood in children aged three to eight years old across a 9-month school year, although it does not include a physical activity component. WISE includes weekly food experiences and supporting activities that align with ECE educational standards and has been shown to create positive changes in both child and family eating behaviors. These include incorporation of more fruit and vegetables into the diet after experiencing WISE and decreased intake of nutrient-poor foods (e.g., chips, cookies, candies) compared to children not exposed to WISE (40, 41). WISE has been disseminated since 2012 and continues to be disseminated primarily in Arkansas, US.

### Participant recruitment

Both FF and WISE maintain databases of previously trained ECE centers, which provided the sampling pool for the survey. In total, the WISE database included 209 centers, and the FF database included 212 centers. All centers in the training databases were eligible for survey participation. Directors from each center were invited to complete the survey *via* email invitation first; these invitations were followed with phone invitations if the email did not receive a response. Our target sample size was 112 (*n*_WISE_ = 49 and *n*_FF_ = 63) to provide 80% power to detect medium sized effects and reflect the imbalance of trained centers in each state to date (17). However, due to recruitment challenges experienced during the COVID-19 pandemic, actual recruitment numbers differed.

Prior to sending email invitations, study staff confirmed email contact information for the site director *via* website or phone call. Each center director received an initial email invitation to the survey. Centers that did not respond to the initial email invitation or two reminder emails were contacted by phone by trained study staff. Data collection took place between January and September 2021.

The survey was divided into sections that assessed general use of nutrition practices at the center (i.e., first portion) and a section that assessed specific use of either FF or WISE (i.e., second portion). Participants had the option to continue to the FF and WISE specific portion of the survey. Only participants who completed the second portion of the survey were included in the following analysis.

### Survey

The survey was divided into 5 sections: (1) Your Role at the Center (2) Nutrition and Physical Activity at the Center, (3) FF/WISE Programming at the Center, (4) Factors Influencing the Use of FF/WISE at the Center, and (5) What It Is Like at the Center. These sections reflected adaptations of three key measures: Steckler's Perception of Innovations (42), the Organizational Readiness for Change Assessment (ORCA) (43), and the Program Sustainability Assessment Tool (PSAT) (44). The Steckler measure, consistent with the DSF construct of Intervention, was chosen to measure attitudes toward the innovations broadly (i.e., nutrition and physical activity) and adapted for each program to measure attitudes about FF/WISE specifically. The ORCA measure captured issues relevant to the DSF construct of Practice Setting (e.g., culture, leadership), and the PSAT captured constructs relevant to both the Practice Setting (e.g., organizational capacity) and the Ecological System (e.g., external environmental support). The complete survey is included in Supplementary materials; the survey was estimated to take 30 to 45 minutes to complete. Participants were asked to think about their center when it was operating normally (before COVID-19). A summary of the survey content is provided in Table 1 including the constructs measured in each section of the survey, the number of items per construct, and relevant reliability and validity information. Correlations between measured variables, mean scores and standard deviations can be found in Table 2.

**Table 1:** Survey content, source, and reliability of all measures included in the director survey.

**Survey section**	**Item content**	**Number of items**	**Source**	**Reliability**
Your role at the center	Participant/Center characteristics	15	Self-developed	NA
Nutrition and physical activity at the center	Continued attention to nutrition/PA at the center	5	Self-developed	α = 0.83
	Concern about nutrition/PA	8	Adapted from Steckler (42) Awareness Concern about Prevention scale placeholder 0	α = 0.61
	Nutrition/PA training	15	Self-developed	NA
	WISE/Food Friends or equivalent use	2	Self-developed	NA
	Use of program resources	7	Self-developed	NA
WISE/Food Friends programming at the center	Program Fidelity	7	WISE fidelity (45) checklist placeholder 1 (adapted and mirrored for Food Friends sites)	
	Level of use	2	Steckler (42) Perceptions of the Innovation.	NA
	Level of success	3	Steckler (42) Perceptions of the Innovation.	NA
	Relative advantage	4	Steckler (42) Perceptions of the Innovation.	α = 0.69 FF, 0.93 WISE
	Level of institutionalization	7	Steckler(42) Perceptions of the Innovation.	α = 0.90 FF, 0.87 WISE
	Environmental support	3	Program Sustainability Assessment Tool (44)	α = 0.91 FF, 0.91 WISE
Factors influencing the use of	Funding and resource stability	3	Program Sustainability Assessment Tool (44)	α = 0.90 FF, 0.87 WISE
WISE/Food Friends at the	Organizational capacity	3	Program Sustainability Assessment Tool (44)	α = 0.96 FF, 0.93 WISE
center	Program adaptation	3	Program Sustainability Assessment Tool (44)	α = 0.91 FF, 0.88 WISE
	Communications	3	Program Sustainability Assessment Tool (44)	α = 0.89 FF, 0.94 WISE
	Strategic planning	3	Program Sustainability Assessment Tool (44)	α = 0.92 FF, 0.95 WISE
	Staff culture	3	Organizational Readiness for Change Assessment (43)	α = 0.83 FF, 0.94 WISE
What it is like at the center	Opinion leaders	3	Organizational Readiness for Change Assessment (43)	α = 0.93 FF, 0.77 WISE
	General resources	4	Organizational Readiness for Change Assessment (43)	α = 0.74 FF, 0.78 WISE
Participant demographics	Gender, age, race, ethnicity	6	US Census Bureau	NA

**Table 2:** Means, standard deviations, and correlations of all variables.

**Variable**	***M***	***SD***	**1**	**2**	**3**	**4**	**5**	**6**	**7**	**8**	**9**	**10**	**11**	**12**	**13**	**14**
1. Program	0.53	0.50														
2. Lag	3.42	2.19	0.43**													
3. Funding stability	3.33	1.98	−0.26	−0.26												
4. Environmental support	3.46	1.78	−0.18	−0.36**	0.77**											
5. Organization capacity	4.25	1.90	−0.03	−0.25	0.79**	0.71**										
6. Program adaptation	3.63	1.81	−0.13	−0.11	0.56**	0.52**	0.66**									
7. Communications	3.25	1.66	−0.13	−0.10	0.73**	0.76**	0.79**	0.69**								
8. Strategic planning	3.29	1.93	−0.14	−0.27	0.86**	0.74**	0.87**	0.66**	0.81**							
9. Level of use	0.93	0.47	−0.05	−0.25	0.19	0.22	0.31	0.33*	0.29	0.36*						
10. Level of success	62.16	12.73	0.12	0.15	0.40*	0.41**	0.42**	0.17	0.43**	0.37*	0.26					
11. Relative advantage	3.28	0.48	−0.09	−0.27*	0.45**	0.50**	0.48**	0.37**	0.44**	0.55**	0.14	0.33*				
12. Level of institutionalization	2.67	0.73	−0.14	−0.12	0.73**	0.72**	0.76**	0.61**	0.72**	0.80**	0.27	0.48**	0.52**			
13. Staff culture	4.50	0.53	0.11	0.07	0.16	0.15	0.28	0.16	0.23	0.28	0.10	0.36*	0.26	0.05		
14. Opinion leader	4.41	0.60	−0.05	−0.14	0.24	0.16	0.39**	0.17	0.22	0.30*	0.24	0.37*	0.27	0.22	0.48**	
15. General resources	3.78	0.70	0.13	0.15	0.27	0.11	0.39**	0.03	0.24	0.22	0.13	−0.06	0.10	0.24	0.19	−0.07

M and SD are used to represent mean and standard deviation, respectively.

^*^Indicates p < 0.05.

^**^Indicates p < 0.01.

#### Director role at the center

In this section, items assessed characteristics of the center and the person completing the survey including: (1) level of involvement in decisions about nutrition and physical activity at the center, (2) years of experience in ECE and at the center, (3) role at the center and years in the role, (4) other roles at the center, (5) whether the program was a Head Start, (6) the center's total capacity and hours of operation, (7) tax status of the center, and (8) school district (if applicable). These items were used to describe survey participants and to screen for eligibility for completing the survey. Individuals with no role in making decisions about nutrition and physical activity at the center were asked to provide an alternate email for the person involved in those decision. At the end of this section, respondents were asked if they wanted to continue the survey.

#### Nutrition and physical activity at the center

Items in this section focused on Continued Attention to Nutrition/PA at the center, Concern about Nutrition/PA, Program Component Usage, and Nutrition/PA Training. Continued Attention is sustaining attention to the issue (i.e., nutrition/PA through policy and/or resource allocation), even if specific programs/interventions are not sustained *per se* (e.g., Rate the level of focus for your program for providing children opportunity to try new or unfamiliar foods). Continued Attention items were self-developed based on salient aspects of the training and evidence-base of FF and WISE (e.g., intentional exposures to new foods). Items on Concern about Nutrition/PA were adapted from the Steckler and colleagues measure of Awareness Concern about Prevention scale (42). Nutrition/PA Training items assessed how frequently in the last 5 years that staff had received training in specific nutrition/PA topics (none to a lot). Nutrition/PA Training items were akin to a checklist, which would preclude internal consistency as an appropriate assessment of reliability. To note, self-developed responses for FF and WISE were developed to capture similar aspects of the programs, and thus the same questions were asked for each program, allowing for data to be aggregated across programs. Sum scores were created for these constructs with higher scores reflecting greater use and training levels.

At the end of this section, participants indicated if they had used FF and WISE in the prior 7 years. Survey items also branched to ask for the number of years the program was used, the most recent year of use of each program, and their role with the program. We also include an open-ended response on reason for discontinuing use. Based on participants' response to the question about use of FF/WISE, the remainder of the survey was specific to their experience with either FF and WISE (i.e., branching logic replaced program names as applicable throughout the remainder of the survey). If the program indicated no use of FF and WISE in the past 7 years, the survey ended.

#### Food Friends/WISE programming at the center

In this section, participants provided responses to items about Program Fidelity, as well as Level of Use, Level of Success, Relative Advantage, and Level of Institutionalization which were adapted items from the Steckler Perception of Innovations Measure (37). Program Fidelity items were designed to mirror that of the published WISE fidelity measure (45) and a corresponding and adapted item set for FF. Items were averaged to get an overall fidelity score. All remaining scales were based on Steckler measures on Perceptions of the Innovation (42). Level of Use included yes/no questions about integration of the programs into routine and standing curriculum. Level of Success included items rated on a sliding scale from Not at All (0) to Completely (100; e.g., The program met your goals). Relative advantage items ask about perceived effectiveness and quality of the programs and were averaged to create a scale score. Finally, the Level of Institutionalization scale assessed factors associated with integrating the programs into center activities (e.g., weekly classroom schedules, overall curriculum) with ratings on a 4-point scale (Strongly Disagree to Strongly Agree). Scale scores were created by averaging across items; for subscale means and standard deviations, see Table 2.

#### Factors influencing the use of Food Friends/WISE at the center

This section included items adapted from the Program Sustainability Assessment Tool (PSAT) (44). Specifically, items were selected and adapted from the constructs of Environmental Support [e.g., FF/WISE had champions or advocates who garnered additional resources (e.g., food, community, donations)], Funding and Resource Stability [e.g., FF/WISE had sustained funding at your center (e.g., food costs, replacement materials)], Organizational Capacity (e.g., Our center had adequate staff to complete FF/WISE goals.), Program Adaptation [e.g., Our center adapted to changes in the environment for FF/WISE (e.g., turnover, leadership change)], Communications (e.g., Our center promoted FF/WISE in a way that generated interest [e.g., wall displays, parent communications)], and Strategic Planning (e.g., Our center had a long-term sustainability plan for FF/WISE beyond our initial year of implementation). Each of these constructs was captured with 3 items each on a 1 (To Little or No Extent) to 7 (To a Very Great Extent) scale.

#### What it is like at the center

The final section of the survey included items from the Organizational Readiness for Change Assessment (ORCA) including items on Staff Culture, Opinion Leaders, and General Resources (43). These questions were rated on a 1 (Strongly Disagree) to 5 (Strongly Agree) scale.

#### Cognitive interviewing

The research team conducted 5 cognitive interviews to refine and adapt survey items, three with prior participants in the FF program and two prior participants in the WISE program. For the interviews, one study Principal Investigator (PI) and one research assistant held a video conference with each participant. The participant opened the survey on their personal computer and shared their screen as they completed the survey. The research team invited the participants to talk aloud as the completed the survey, explain the rational for their responses, ask questions about the items and/or instructions, and note any aspects that were confusing or unclear. The researchers documented the items on which participants had comments and questions and asked the participants to suggest improved wording. In addition, the researchers prompted the participants at the end of each page to give feedback about the format, item response options, and instructions. The researchers also monitored for signs of confusion (e.g., excess scrolling, mouse movements) to prompt participants to explain their thought processes. Finally, the researchers asked participants to review the initial survey instructions both before and after completing the survey to improve clarity about the survey's purpose and contents. Improvements were made to the survey iteratively to test changes in wording with subsequent interview participants. The PSAT Partnership and Program Evaluation sub-scales were excluded from the full survey because of confusion and poor performance during cognitive interviews.

### Analyses

#### Careless responding

Four measures were used to investigate levels of careless responding to identify problem cases in the data: Mahalanobis Distance, long-string analysis, survey duration, and even-odd consistency (46). If a participant response was flagged under at least two of the above conditions, their responses were investigated for concordant responding (e.g., their responses to conceptually similar items were checked for consistent responses). Mahalanobis Distance is a measure of multivariate normality. Participant response sets (i.e., their pattern of responses to every survey question) were compared to the average response set using a Mahalanobis Distance value, and *p*-values were generated identifying participants whose response sets were multivariate outliers. Long-string analysis looks for consistent identical responding within surveys (e.g., selecting “Slightly Agree” for ten items in a row), and acceptable cut-off values are determined based on survey design. An even-odd consistency correlation can assess the extent to which participants chose similar answers to even and odd questions within a given survey, with inconsistent responses indicated by low correlation scores (see Supplementary materials for more information on this process).

#### Program sustainment

The key outcomes of program sustainability were conceptualized in two ways: Continued Practice (i.e., the use of or general focus on nutrition programs or PA programs at the center) and Program Fidelity (i.e., how well centers used specific evidence-based practices of FF or WISE). Continued Practice was calculated by summing up scores from four measures, described in the Nutrition and Physical Activity at the Center portion of the survey, that capture the extent to which program elements were being used at centers. These included a measure of continued attention to nutrition and PA at the center (e.g., “Rate the level of focus for your program: teaching children about nutrition”), concern about nutrition and physical activity at the center (e.g., “How true are the following statements at your center?: I am concerned with the level of activity children get”), the use of nutrition program components (e.g., “How often do children at your site engage in the following activities: Teacher/adult-led physical activities during outdoor play (like recess)”), and nutrition and PA training (e.g., “How much training content have staff at your center received in the following: portion sizes for children; creating positive mealtimes”). From these items, composite scores for Nutrition Continued Practice and Physical Activity Continued Practice were calculated separately. FF consisted of two programs that were targeted at changing nutrition and physical activity practices in ECE contexts, whereas WISE is only targeting nutrition in ECE. Therefore, central comparisons of continued practice are made on the continued practice of nutrition, and physical activity continued practice is a secondary variable/outcome measured only for FF.

#### Program Fidelity

Program Fidelity was calculated by a set of seven items that measure the extent to which each center was following key components of FF or WISE. Participants indicated the extent to which their centers were using key elements of FF or WISE in the last year each program was implemented (e.g., “Used the Food Friends puppets and characters with the lessons” or “Used the Windy Wise mascot with WISE lessons). Responses to the seven items are summed to create the Program Fidelity score.

#### Analysis plan

The original analysis plan for this survey data indicated that measures of sustainability would be determined based on continued practice and attention to best practices in nutrition education and adherence to specific program elements. Responses to the PSAT, Steckler Perceptions of Innovation, and the ORCA would be used as predictors of these measures of sustainability, as well as the interaction between the subscales of the PSAT measure and lag. The previous analysis plan (17) was altered due to two main factors that emerged from the current data set: lower than anticipated sample size and high intercorrelation between theorized predictors of sustainment. Due to our final sample of *N* = 55, regression analysis with the initial number of predictors (all subscales of each measure, lag, program, and interaction between lag and PSAT subscales) were no longer adequately powered. Additionally, both the adapted PSAT (α = 0.97) and Steckler Perception of Innovation (α = 0.89) measures had high internal consistency across items regardless of subscale. When correlations between subscales were investigated, the intercorrelations among subscales within these scales caused substantial multicollinearity issues (i.e., VIF values > 5 and reversal of the direction of bivariate correlations directions vs. beta-weights, see Table 2 for correlations of all variables used in current analysis). For instance, the six subscales of the PSAT measure had intercorrelations ranging from *r* = 0.51 to *r* = 0.87.

Therefore, it was determined multiple regression models with sustainment variables as outcomes, and the overall average scores of the PSAT, Steckler Perceptions of Innovations, and ORCA subscales, program type, and lag entered as predictors would be used to determine which overall measures were predictive of sustainment outcomes. Following these regressions, any overall scale that was predictive of a sustainment outcome would be investigated further by looking at the bivariate correlation between corresponding subscales and the sustainment outcome. Distribution of scores for all subscales and overall measures were investigated to determine if there were significant outlier scores or issues with normality. There were no individual averages for the PSAT, Steckler, or ORCA that were greater than three standard deviations away from the mean, and the Mahalanobis Distance analysis described above to investigate careless responding did not identify multivariate outliers among participant response sets. Program (FF or WISE) differences in PSAT, Steckler Perceptions of Innovations, and ORCA subscales were also assessed using MANOVAs in order to determine if there were program-specific differences in these variables. All analyses were conducted in SPSS 27 (Windows, Version 27.0. Armonk, NY: IBM Corp).

## Results

### Sample demographics

A total of 105 participants (*n* = 51 WISE participants, *n* = 54 FF participants) began the survey. Of the 105 individuals that began the survey, 82 (78%) completed the first portion of the study, 58 (55%) proceeded to the end, and three were later removed from the sample due to careless responding. Thus, there were a total of 55 participants (*n*_*WISE*_ = 26, *n*_*FF*_= 29) whose responses about their centers were included in the final analysis. Most participants were female (*n* = 52, 94.5%), White (*n* = 48, 87.3%; Black = 4, 7.3%; American Indian or Alaskan Native = 1, 1.8%; missing = 2, 3.6%) and non-Hispanic (*n* = 51, 92.3%; Hispanic = 3, 5.5%; missing = 1, 1.8%). The average participant age was 49.3 years (*SD* = 8.9, minimum (min) = 32 years, maximum (max) = 65 plus years; missing = 19). Participants had worked an average of 21.0 years in ECE (*SD* = 9.4, min = 32 years, max = 45 years; missing = 2) and had worked at their current center for an average of 14.0 years (*SD* = 8.2, min = 4 years, max = 37 years; missing = 2). Most participants had been in their current role for 0-5 years (*n* = 16, 29%). Most of the participating centers were not Head Starts *n* = 40 (72.7%); served fewer than 100 children; were open 4 or 5 days a week; and were mainly metropolitan (> 50,000 population) and micropolitan (10,000-50,000 population), as determined by U.S. Department of Agriculture Rural-Urban Community Area codes (47). See Table 3 for a breakdown of center-level demographics by state.

**Table 3:** Center–level demographics (*N* = 55) for WISE (*n* = 26) and Food Friends centers (*n* = 29).

	**Overall** **(*N*, %)**	**Food Friends** **(*n*, %)**	**WISE** **(*n*, %)**
**Type of program**			
Head Start	13 (23.6%)	4 (13.8%)	9 (34.6%)
Non–Head Start	40 (72.7%)	25 (86.2%)	15 (57.7%)
Missing	2 (3.6%)	0 (0%)	2 (7.7%)
**Number of children served**			
1–25 children	12 (21.8%)	6 (20.7%)	6 (23.1%)
26–50 children	10 (18.2%)	5 (17.2%)	5 (19.2%)
51–100 children	17 (30.9)	12 (41.4%)	5 (19.2%)
101–200 children	9 (16.4%)	4 (13.8%)	5 (19.2%)
Over 200 children	5 (9.1%)	2 (6.9%)	3 (11.5%)
Missing	2 (3.6%)	0 (0%)	2 (7.7%)
**Number of hours open**		
6 h or less per day	3 (5.5%)	1 (3.4%)	2 (7.7%)
7 to 12 h per day	49 (89.1%)	27 (93.1%)	22 (84.6%)
13 to 18 h per day	1 (1.8%)	1 (3.4%)	0 (0%)
Missing	2 (3.6%)	0 (0%)	2 (7.7%)
**Days per week center is open**			
2 days	1 (1.8%)	1 (3.4%)	0 (0%)
3 days	0 (0%)	0 (0%)	0 (0%)
4 days	11 (20.0%)	9 (31.0%)	2 (7.7%)
5 days	41 (74.5%)	19 (65.5%)	22 (92.3%)
Missing	2 (3.6%)	0 (0%)	2 (7.7%)
**Tax status**			
Non–profit	39 (70.9%)	21 (72.4%)	18 (69.2%)
For–profit	7 (12.7%)	5 (17.2%)	2 (7.7%)
Don't Know	7 (12.7%)	3 (10.3%)	4 (15.4%)
Missing	2 (3.6%)	0 (05)	2 (7.7%)
**USDA rural–urban community area classification**			
Metropolitan	16 (29.1%)	8 (27.6%)	8 (30.8%)
Micropolitan	16 (29.1%)	6 (20.7%)	10 (38.5%)
Small town to rural	9 (16.4%)	7 (24.1%)	2 (7.7%)
Missing	14 (25.5%)	8 (27.6%)	6 (23.1%)

Lag was determined as the number of years since FF or WISE has been implemented at a center. For WISE centers, the mean number of years it had been since implementation was 1.4 years (*SD* = 1.3 years, min: 0 years, max: 6 years). For FF centers, the mean number of years since last implementation was 3.3 years (*SD* = 2.4 years, min = 0 years, max = 8 years). The mean difference in lag between FF and WISE was significant (*t*(53)= 3.51, *p* < 0.001). Chi-square tests did not indicate that center demographics or director demographics were significantly associated with survey completion.

### Program differences in predictors of sustainment

Two-way MANOVAs were used to investigate if PSAT, Steckler Perceptions of Innovations, and ORCA subscale measures differed by program type (FF/WISE) after controlling for lag. There were no differences in PSAT scores by program (*F*_(6, 33)_ = 2.05, *p* = 0.087, $\eta_{\text{p}}^{2}$ = 0.27). Neither the Steckler Perceptions of Innovations (*F*_(4, 28)_ = 1.33, *p* = 0.285, $\eta_{\text{p}}^{2}$ = 0.16) or ORCA (*F*_(3, 43)_ = 0.195, *p* = 0.899, $\eta_{\text{p}}^{2}$ = 0.013) subscales differed significantly by program.

#### Predictors of sustainment continued practice

The regression model with program, lag, overall ORCA, Steckler Perception of Innovation, and PSAT scores accounted for a significant proportion of variance in Nutrition Continued Practice scores (*F*_(5, 45)_ = 4.13, *p* = 0.004, *R*^2^ = 0.24; see Table 4). Program was a significant predictor of Nutrition Continued Practice scores (β = −0.32, *t* = −2.28, *p* = 0.028). WISE programs reported higher Nutrition Continued Practice (*M* = 11.47, *SD* = 1.83) compared to FF programs (*M* = 10.27, *SD* = 2.13). Overall PSAT score was also a significant predictor of Nutrition Continued Practice (β =0.423, *t* = 3.11, *p* = 0.003). Because of issues with multicollinearity among PSAT subscales, follow-up analyses looking at the relationship between PSAT subscales and Nutrition Continued Practice were performed with simple bivariate correlations. Nutrition Continued Practice was significantly positively correlated with all PSAT subscales: communications (*r* = 0.51, *p* < 0.001), funding stability (*r* = 0.49, *p* < 0.001), strategic planning (*r* = 0.45, *p* < 0.001), organizational capacity (*r* = 0.43, *p* = 0.001), environmental support (*r* = 0.39, *p* = 0.004), and program adaptation (*r* = 0.34, *p* = 0.01). The regression model with program, lag, and overall ORCA, Steckler Perception of Innovation, and PSAT scores did not predict a significant portion of variance in FF-only Physical Activity Continued Practice scores (*F*_(4, 22)_ = 0.28, *p* = 0.89, *R*^2^ = 0.05).

**Table 4:** Results of regression models predicting sustainment outcomes (Nutrition continued capitalization is inconsistent practice, physical activity continued practice, and program fidelity).

**Sustainment outcome**	***t***	***p***	**β**	***F***	***df***	***p***	**adj. R^2^**
**Nutrition Continued Practice**							
Overall Model				4.13	5, 45	0.004	0.24
Program	−2.28	0.03	−0.32				
Lag	0.21	0.16	0.21				
PSAT	3.11	0.003	0.42				
Steckler Perception of Innovations	0.51	0.61	0.07				
ORCA	0.84	0.40	0.11				
**Physical activity continued practice (CO Only)**							
Overall model				0.28	4, 22	0.89	0.05
Lag	−0.68	0.50	−0.15				
PSAT	0.55	0.59	0.13				
Steckler Perception of Innovations	0.07	0.95	0.014				
ORCA	−0.40	0.69	−0.09				
**Program Fidelity**							
Overall model							
Program	0.63	0.53	0.07	13.31	5,45	< 0.001	0.55
Lag	−1.21	0.23	−0.14				
PSAT	6.00	< 0.001	0.63				
Steckler Perception of Innovations	2.10	0.04	0.22				
ORCA	0.61	0.54	0.06				

#### Program Fidelity

The regression model predicting Program Fidelity indicated that program, lag, and overall ORCA, Steckler Perception of Innovation, and PSAT scores accounted for a significant amount of variance in Program Fidelity scores (*F*_(5, 45)_ = 13.31, *p* < 0.001, *R*^2^ = 0.55). Both the overall PSAT score (β = 0.626, *t* = 6.00, *p* < 0.001) and overall Steckler Perception of Innovation (β = 0.219, *t* = 2.10, *p* = 0.041) were significant, positive predictors of Program Fidelity scores. Program Fidelity scores were significantly and positively correlated with all PSAT subscales: organizational capacity (*r* = 0.73, *p* < 0.001), program adaptation (*r* = 0.66, *p* < 0.001), communications (*r* = 0.66, *p* < 0.001), strategic planning (*r* = 0.58, *p* < 0.001), environmental support (*r* = 0.57, *p* < 0.001), and funding stability (*r* = 0.46, *p* < 0.001). Program Fidelity was significantly correlated with only two of the four Steckler Perceptions of Innovation measures: level of institutionalization (*r* = 0.61, *p* < *0*.001) and relative advantage (*r* = 0.54, *p* < *0*.001).

## Discussion

This study contributes to the limited literature on sustainment of nutrition/PA programs in ECE (25) by examining predictors of sustainment across two nutrition/PA programs in two U.S. locations. Specifically, we examined how indicators of the Dynamic Sustainability Framework constructs were associated with sustainment in the presence of other DSF constructs, answering recent calls to use theory to evaluate the sustainment of interventions (30). Specifically, our study was able to identify evidence to support the importance of each DSF construct in understanding sustainment, both for sustaining attention to nutrition/PA broadly and to sustaining the programs as designed. Overall, our data suggest that contextual and system factors may be more important for sustainment than characteristics of the intervention.

For the construct of Intervention, perceptions of the innovation were a significant predictor of sustained Program Fidelity but not Continued Attention (either nutrition on PA), providing evidence that program-specific attitudes influence program-specific outcomes. The Steckler constructs of Institutionalization and Relative Advantage were most highly associated with sustained Program Fidelity. That is, perceiving FF/WISE as better than alternative program options and integrating FF/WISE into center schedules, routines, and norms was correlated with programs' continued use of specific program elements (i.e., Program Fidelity). This finding is consistent with a recent review finding perceived benefits and program integration as key factors for sustained implementation of health behavior programs in schools and ECEs (25). It is also consistent with qualitative research on sustaining IMIL in ECE settings, which identified integration into the curriculum and routine as key for sustainment (35).

We also examined program differences in outcomes to further examine the association of Intervention characteristics with sustainment outcomes. Only one difference between FF/WISE programs was observed; Nutrition Continued Practice was significantly higher for WISE compared to FF after controlling for lag and other predictors. This may be because of the singular focus of WISE on nutrition compared to the dual focus of FF on nutrition and PA. For example, Ward and colleagues found that ECE centers were more likely to maintain healthy eating than physical activity components of their intervention, stating that focusing on both may be a challenge for centers (35). Overall, the similarities in findings for FF/WISE suggest either true overlap in sustainment related outcomes and predictors despite the program type, lack of power to detect differences, or similarities due to measurement characteristics. Future in-depth qualitative research will explore these possibilities.

Beyond the DSF construct of Intervention, some findings support the association of the Practice Setting and Ecological System with sustainment outcomes. In fact, the overall PSAT score was the most important predictor in the presence of other predictors for both outcomes. Specifically, both Nutrition Continued Practice and Program Fidelity were significantly predicted by overall PSAT scores with high correlations will all PSAT sub-scores. Indicators of the importance of the Practice Setting included moderate to strong correlations between sustainment outcomes and communication, strategic planning, the center's adaptation of programs, and organizational capacity. While communications and planning are potentially malleable targets for supporting sustainment, organizational capacity may be less so. Consistent with a 2020 review by Herlitz et al. of sustainment of public health programming in schools (34), our study suggests that some organizations may be disadvantaged from the outset for achieving sustainment. Specifically, program capacity was an important predicator of sustainability across both programs and both targeted outcomes, consistent with the importance of capacity in prior reviews of sustainment of community-based public health interventions (48) and of health behavior interventions in schools and ECE settings (25). Prior research has also suggested that adaptation to the local context is key for sustainment of a program as well as sustained impact if fidelity to components are maintained (48). The self-report nature of our study did not allow us to determine if adaptations were fidelity consistent or inconsistent. In-depth observations at study sites in subsequent research will shed light on this issue. Despite these indicators of the importance of the practice setting, organizational readiness (as measured by the ORCA) was not related to either sustainment outcome in the presence of other predictors in our sample. This is counter to a recent review of health behavior interventions in schools and ECE settings (25), which found organizational readiness to be among the most frequently identified inner context factors important for sustainment.

The importance of the Ecological System was supported with a strong correlation between PSAT Environmental Support and Program Fidelity, a moderate association between PSAT Environmental Support and Nutrition Continued Practice, and moderate associations between PSAT Funding Stability and Program Fidelity and Nutrition Continued Practice. Our findings on the importance of funding are consistent with a review of studies on sustainment of obesity prevention programs in community settings, which identified resources as the most frequently identified factor for sustainment (24). Shoesmith et al. also identified funding availability as the most frequently cited outer context barrier to sustainment in their review of school and ECE-based health behavior interventions (25). Funding stability for an ECE program may have direct impact on use of a nutrition/PA program (e.g., purchase of supplies) or indirect impact (e.g., under-staffed, under-resourced work climates). Future research should explore these potential mechanisms. Our data suggest that support beyond funding is also needed. Although our study did not examine nuance in types of environmental support, prior research has identified parent engagement as key to sustainment in the ECE setting (35). Center leadership and teachers may benefit from an external “pull” from parents to provide this type of programming. Sustainment strategies targeting the ECE Ecological System are limited in the literature and may have value.

Taken together, these results support the importance of all levels of the DSF in understanding sustainment. Specifically, intervention characteristics (e.g., program type, perceptions of innovation), practice setting traits (e.g., organizational capacity, communications), and the ecological system (e.g., environmental support) were important predictors in our study. Although not tested in our study, elements identified by the DSF may be interlinked in complex manners. For example, evidence-based practice integration and continued training over time have been identified as important predictors of sustainment (25, 33), but these activities are more difficult to implement for institutions where financial stability and staffing constraints are more prominent, perhaps linking certain sustainment predictors together *via* institutional revenue and monetary resources. We were not able to test interactions as expected because of challenges with measuring factors related to sustainment.

### Challenges, limitations, and strengths

The primary challenge we faced in measurement were high intercorrelations between sub-scales of the PSAT in our sample. Specifically, all sub-scales were correlated at or beyond *r* = 0.52, contributing to high variance inflation factors in the proposed analyses and a need for a revised analysis approach. This was a somewhat unexpected finding because original confirmatory factor analyses of the PSAT in over 250 public health programs (e.g., tobacco control, diabetes prevention) supported a factor structure with 8 distinct domains (44). However, a recent examination of the PSAT in school settings demonstrated an overarching Cronbach's alpha for internal consistency of 0.95 (33), suggesting high overlap between scales much like our sample. Together with the findings of our study, data suggest that the PSAT may need further revision and testing to have appropriate discriminant validity between sub-scales for educational settings. Further, the lack of association between the ORCA constructs and outcomes in our study may suggest need for further measure development/adaptation around organizational readiness for the ECE setting. In future work, a sufficiently powered sample could be used to perform confirmatory factor analyses (CFA) and invariance testing to establish similar performance over various samples for these measures.

The study has additional limitations and strengths. A key limitation is that study recruitment and data collection was conducted during the COVID-19 pandemic. Programs that were able to be reached and participate during this time may differ in systematic ways from programs that were non-responsive. Specifically, it is possible that only more resourced and/or engaged centers were able to respond, which may have truncated the range of variables in our study. This concern is somewhat attenuated by the findings on program capacity's influence on sustainment outcomes, which indicates useful variability was present in the sample. A related limitation is that our sample size did not reach desired numbers for the previously proposed moderation analysis. Based on initial recruitment predictions, it was estimated that approximately 40% of the potential recruitment pool would respond to the director survey (*n* = 150; WISE programs = 45, FF programs = 105). We did not reach these numbers, and many programs that started the survey did not complete it in its entirety (45%). Thus, our study was slightly under-powered compared to our original design. Several strengths offset these limitations. First, we were able to collect information about two distinct programs across two U.S. locations. This increases the generalizability of our findings about the key factors associated with sustaining nutrition/PA programs in ECE. We were also able to model wide variation in lag since implementation, despite surprising null findings regarding its predictive power. Finally, our study was able to simultaneously examine multiple domains theorized by the DSF to be associated with sustainment outcomes in an ECE setting. This approach revealed that, for the present sample, contextual and systems characteristics were the most predictive of continued attention to nutrition/PA and specific program practices.

### Implications for future research and practice

Similar to prior systematic reviews (49), our results indicated that organizational capacity and centers' adaptation of programs were strongly correlated with Program Fidelity. Targeted capacity building and intentional local adaptation during the pre-implementation phase may better prepare programs to self-sustain evidence-based practices over time. Partnered approaches to building local capacity are emerging as examples to inform further research in this area (33, 50, 51). Future research could explore the value of sustainment strategies targeting contextual factors in the pre-implementation and implementation phases for long-term outcomes. Implementation practitioners may see more benefit from advocating for systems changes and addressing contextual challenges than working directly with implementers and the innovation. Additionally, intentional efforts to support centers as they adapt programs may support long-term fidelity and sustainment.

In the presence of a supportive system and stable context or adjacent to addressing these factors, our data particularly support the importance of local perceptions of innovation as an area for future research and practice. In our study, perceiving FF or WISE as being better or more advantageous than other alternatives was related to higher Program Fidelity. Future research could explore the unique value of sustainment strategies that target adopter perceptions of innovations as well as technical assistance or facilitation approaches that provide structured support for ECE centers to integrate innovations into their program goals and schedule, both at the outset and as an ongoing effort. Practitioners may support implementers by directly addressing their thoughts, attitudes, and motivations related to the targeted innovation. These factors should be considered from the outset of program development and initial training.

## Conclusions

Our study supports the importance of each DSF construct in understanding sustainment, both for sustaining attention to nutrition/PA broadly and to sustaining the programs as designed. Further, our data demonstrate that contextual and system factors may be more important for sustainment than characteristics of the intervention. This study also suggests that factors associated with the continued practice of program principles are partially distinct from those that are associated with the sustainment of specific practices driving program fidelity. Thus, capacity building strategies may be important for both continued attention to nutrition and PA as well as sustaining fidelity to specific evidence-based practices.

## Data availability statement

The raw data supporting the conclusions of this article will be made available by the authors, without undue reservation.

## Ethics statement

The studies involving human participants were reviewed and approved by UAMS Institutional Review Board, University of Arkansas for Medical Sciences. Written informed consent for participation was not required for this study in accordance with the National Legislation and the Institutional Requirements.

## Author contributions

TS led the drafting and revision of this manuscript. TS and LB led the conception and design of this study in addition to obtaining funding. GC conducted the analyses for the study, contributed to drafting the manuscript, and coordinated the initial submission of this manuscript. DZ contributed to the drafting and editing of this manuscript. SJ contributed to the design of this study and editing of this manuscript. GC and JS contributed to the conception and design of the study and editing of this manuscript. All authors approved the manuscript before submission.

## Funding

The project was funded by NIH R21CA237984 (TS and LB). TS was also supported by the National Institute of General Medical Sciences of the National Institutes of Health under Award Number 5P20GM109096. TS and GC have salary support from NIH UL1 TR003107. TS, GC, DZ, VM, SJ, and JS have salary support from R37CA252113.

## Conflict of interest

Authors LW-M and TS have a financial interest in the technology (WISE) discussed in this presentation/publication. These financial interests have been reviewed and approved in accordance with the UAMS conflict of interest policies. The remaining authors declare that the research was conducted in the absence of any commercial or financial relationships that could be construed as a potential conflict of interest.

## Publisher's note

All claims expressed in this article are solely those of the authors and do not necessarily represent those of their affiliated organizations, or those of the publisher, the editors and the reviewers. Any product that may be evaluated in this article, or claim that may be made by its manufacturer, is not guaranteed or endorsed by the publisher.

## Author disclaimer

The content is solely the responsibility of the authors and does not necessarily represent the official views of the funding agencies.
