# Supplementary material for: Predictors of sustainment of two distinct nutrition and physical activity programs in early care and education
Source: Front Health Serv. 2022 Nov 9;2:1010305. doi: 10.3389/frhs.2022.1010305 (PMC10012648; doi:10.3389/frhs.2022.1010305)
Supplement: Supplementary file 1 [file Data_Sheet_1.docx]

Supplementary Material

- 1. **Indicators of Careless Responding**

Four measures were used to investigate levels of careless responding to identify problem cases in the data: Mahalanobis Distance, Longstring analysis, survey duration, and Even-Odd Consistency. If a participant’s response set met two of the four criteria, their response was flagged for review by the research team.

Mahalanobis Distance is a measure of multivariate normality. Three different MD estimates were used to identify outliers. The M-distance was calculated for the director survey item responses, based on the director survey responses for participants who also completed the WISE/FF survey, once for the WISE survey item responses, and once for the FF survey item responses. The M-distance for each participant is then compared to a chi-square distribution (degrees of freedom based on the number of items used to calculate M-distance), and significant values indicate that the participant’s response set deviates significantly from the average response set. Here, participants were flagged under two conditions: M-distance exceeded critical value of *p* = .01 and critical value of *p* = .001. No participants were flagged under either of these conditions.

Longstring analysis identifies repetitive participant response patterns (e.g., the participants who select only “neutral” for a list of ten opinion statements). Longstring analysis was approached in two different ways. Because the response anchors varied by measure and the sequence of potential repetition varied based on survey design (e.g., the order the items were presented in and the number of items per ‘page’ of the survey), ten longstring values were calculated for each participant, with values ranging from 4 to 18, depending on how many scale items with the same response options it was possible to repeat. It was determined that the critical value for longstring cutoff would be 7 identical sequential responses. This number was based on the median number of potential identical sequences for each segment of the survey. Of the 59 participants who provided some data for the WISE and FF surveys, 42 (71.2%) participants had at least one longstring value greater than or equal to 7.

After looking at the results of this approach, it was determined that it might be too punitive. For instance, some participants who exceeded the threshold value showed repeated answers on a single, longer measure and low levels of identical responding on all other nine measures. To investigate repeated identical responding further, we considered participants who answered multiple scales (i.e., strings) consecutively with identical values. For example, rather than considering only the length of the string of repeated values, here we considered participants who straightlined multiple scales in a row, rather than just straighlining within scales. Using a critical threshold of three scales with 100% identical responses, only six participants exceeded the threshold of repeating responses over multiple scales. When four was used as the threshold to identify identical responders, only five participants were flagged. Only one participant had identical values across more than four scales in a row with six out of ten strings having 100% identical responses in a row.

Survey duration was also used to flag participants who may have been responding carelessly. Participants who spent than less than five minutes on the second arm of the scale were flagged as potentially careless responders due to the length of the second arm. Of the 59 participants who completed the second arm, only four participants spent less than five minutes completing this arm of the survey.

An even-odd consistency correlation can assess the extent to which participants chose similar answers to even and odd questions within a given survey. For unidimensional surveys, respondent answers to even and odd questions (accounting for reverse-coded items), should be highly positively correlated. If the even-odd consistency correlation is small or negative, this could be an indicator that a participant was carelessly responding. For each scale, two new scores were calculated for each participant: one that was created by averaging responses to even scale items and one that was created by averaging odd scale responses. A within-participant correlation was then calculated to determine the correlation between even and odd scales. Correlations below -.1 were flagged as careless responders. One participant was flagged under this condition (see histogram of Even-Odd Correlation values below). Although this participant was not flagged under the MD calculation, the *p* value associated with their response set for the director survey was *p* = .05 and this participant did not complete the WISE or FF survey.

**Flagged Cases**

Three participants were flagged under at least two conditions described above, and all three were flagged for spending less than five minutes on the second arm of the study and for identical repeated responses across survey measures. Following this procedure, each measure in the survey was reviewed to determine if some levels of consistent responding were more likely or reasonable based on the scale content and if repeated responses were conceptually consistent across survey measures. For example, multiple questions assessed how well participant’s thought FF and WISE were integrated into the curriculum and routines at the participant’s center. Non-concordant answers on similar questions across surveys was considered as an indicator of careless responding. Based on these evaluations, it was determined that two of the three cases identified for the above reasons should be considered careless responders and they were excluded from further analysis.

**1.2 Director Survey**

**________YOUR ROLE AT YOUR CENTER_______**

*This section of the survey will ask you questions about your role at the center. We are interested in getting feedback from the person at your site with the most experience and knowledge about your programming related to nutrition and physical activity*.

|  | **Not at All** | **Somewhat** | **Quite a Bit** | **Very Much** |
| --- | --- | --- | --- | --- |
| 1. I am involved in making decisions about nutrition and physical activity programming and training at my site. (e.g., what trainings staff receive, which curricula we use, assigning resources/staff) | **O** | **O** | **O** | **O** |

1a. Since you selected "not at all" on the involvement question, could you provide an email address for someone else at your center who has been involved in nutrition and PA training? Please enter that person's email. _________________

1. How many years have you worked in early care and education settings? *Please indicate the number of years.*
2. How long have you worked at this center? *Please indicate the number of years.*
3. What is your primary role at the center?

| O | Director/Program Manager | O | Education Coordinator | O | Nutrition Coordinator | O | Other:_________ |
| --- | --- | --- | --- | --- | --- | --- | --- |

1. How many years have you been in this role? *Please select the number of years.*

6. What other roles have you had at this center? *Check all that apply.*

| O | Teacher/Assistant Teacher | | O | Education Coordinator | O | Nutrition Coordinator | O | Other:_________ |
| --- | --- | --- | --- | --- | --- | --- | --- | --- |
| O | | Director/Program Manager | | O | Family Service Coordinator | | | |

7. Is your program a Head Start ?

| O | Yes | O | No |
| --- | --- | --- | --- |

8. What is your center’s total capacity?

| O | 1-25 children | O | 25-50 children | O | 51-100 children | O | 101-200 children | O | Over 200 children |
| --- | --- | --- | --- | --- | --- | --- | --- | --- | --- |

9. What are your hours of operation? Include all hours children are present.

| O | 6 hours or less per day | O | 7 to 12 hours per day | O | 13 to 18 hours per day | O | 24 hours per day |
| --- | --- | --- | --- | --- | --- | --- | --- |

10. How many days per week is your center open?

| O | 1 day | O | 2 days | O | 3 days | O | 4 days | O | 5 days | O | 6 days | O | 7 days |
| --- | --- | --- | --- | --- | --- | --- | --- | --- | --- | --- | --- | --- | --- |

11. What it the tax status of your center?

| O | Non-profit (501c3) | O | For profit | O | Don’t Know |
| --- | --- | --- | --- | --- | --- |

12. What is your center/school district name (classroom if applicable): ________________________

13. The remaining questions on the survey ask about Nutrition and Physical Activity Curriculum at your center. Do you want to continue with the survey? O Yes O No

*(If no)* Why are you stopping the survey? **Open comment box*

**______NUTRITION AND PHYSICAL ACTIVITY AT YOUR CENTER_______**

*This section of the survey will ask questions about your center’s curricular and training practices. Questions will focus on your interest and practices in nutrition and physical activity programing.*

*********Please think about your center when it was operating normally, before COVID19.********

| 1. **Think about how often you introduce new academic curriculum at your center (e.g., literacy, math).** | **Much Less** | **Less** | **The same** | **More** | **Much More** | **Don’t Know** |
| --- | --- | --- | --- | --- | --- | --- |
| How often do you introduce new curricula in Health and Physical Development (e.g., nutiriton, activity) compared to these other areas? | **O** | **O** | **O** | **O** | **O** | **O** |

| 1. **For each topic below, rate the level of focus for your program.** | **Not a Focus** | **A Little Focus** | **Some Focus** | **A Substantial Focus** |
| --- | --- | --- | --- | --- |
| 1. Physical activity for children | O | O | O | O |
| 1. Teaching children about nutrition | O | O | O | O |
| 1. Providing children opportunities to try new or unfamiliar foods | O | O | O | O |
| 1. Development of movement (gross motor) skills | O | O | O | O |
| 1. Preventing child obesity | O | O | O | O |

| **16. How often do children at your site engage in the following physical activities?** | **Less than 1 time per month** | **1- 3 times per month** | **1 time per week** | **2 times per week** | **3 times per week** | | **4- 5 times per week** |
| --- | --- | --- | --- | --- | --- | --- | --- |
| 1. Teacher-led physical activities in the classroom (e.g., music and movement). | O | O | O | O | O | | O |
| 1. Planned movement activities as part of centers, zones, reading, or other individual/small group opportunities. | O | O | O | O | O | | O |
| 1. Teacher/adult-led physical activities during outdoor play (like recess) | O | O | O | O | O | | O |
| 1. Education about physical activity in parent communications (e.g., newsletters, app updates, texts). | O | O | O | O | O | | O |
| **17. How often do children at your site engage in the following nutrition activities?** | **Less than 1 time per month** | **1- 3 times per month** | **1 time per week** | **2 times per week** | | **3 times per week** | **4- 5 times per week** |
| 1. Nutrition education lessons in the classroom | O | O | O | O | | O | O |
| 1. Taste testing of fresh food (e.g., fruits and vegetables) during lessons (not meals or snacks) | O | O | O | O | | O | O |
| 1. Sensory exploration of fresh food (e.g., smelling, observing, touching, interacting with fruits and vegetables with tasting optional) | O | O | O | O | | O | O |
| 1. Nutrition education as part of centers, zones, reading, or other individual/small group opportunities. | O | O | O | O | | O | O |
| 1. Education about nutrition in parent communications (e.g., newsletters, app updates, texts). | O | O | O | O | | O | O |

| 1. **In the last 5 years,** how much training content have staff at your center received in the following: | **None** | **Some** | **A lot** | **I don’t know** |
| --- | --- | --- | --- | --- |
| **Nutrition** |  |  |  |  |
| 1. Portion sizes for children. | **O** | **O** | **O** | **O** |
| 1. Role modeling for children at meals, snacks, or food experiences. | **O** | **O** | **O** | **O** |
| 1. Supporting children to try new foods. | **O** | **O** | **O** | **O** |
| 1. Helping children learn about hunger and fullness signals. | **O** | **O** | **O** | **O** |
| 1. Avoiding pressuring children to eat at meals. | **O** | **O** | **O** | **O** |
| 1. Dietary guidelines (e.g., MyPlate). | **O** | **O** | **O** | **O** |
| 1. Creating positive mealtimes. | **O** | **O** | **O** | **O** |
| 1. Food safety (e.g., temperature, contaminants). | **O** | **O** | **O** | **O** |
| 1. Family style meal service. | **O** | **O** | **O** | **O** |
| **Physical Activity** |  |  |  |  |
| 1. Physical activity guidelines. | **O** | **O** | **O** | **O** |
| 1. Offering physical activity opportunities in the classrooms. | **O** | **O** | **O** | **O** |
| 1. Developing gross motor skills. | **O** | **O** | **O** | **O** |
| 1. Developmentally appropriate physical activity. | **O** | **O** | **O** | **O** |
| 1. Playground safety. | **O** | **O** | **O** | **O** |
| 1. Incorporating physical activity throughout the day. | **O** | **O** | **O** | **O** |

| 1. How true are the following statements for you at your center? | **Not at All True** | **Slightly True** | **Somewhat True** | **Very True** |
| --- | --- | --- | --- | --- |
| 1. I believe promoting healthy habits among children is important (e.g., nutrition, physical activity). | **O** | **O** | **O** | **O** |
| 1. I don’t know why preventing obesity is important for early education settings to address. | **O** | **O** | **O** | **O** |
| 1. I am concerned about children’s eating habits. | **O** | **O** | **O** | **O** |
| 1. I am concerned with the level of activity children get. | **O** | **O** | **O** | **O** |
| 1. I believe children develop gross motor skills without guidance from adults. | **O** | **O** | **O** | **O** |
| 1. I believe children develop healthy eating habits without guidance from adults. | **O** | **O** | **O** | **O** |
| 1. I believe parents are more influential than teachers in helping children develop health eating habits. | **O** | **O** | **O** | **O** |
| 1. I believe parents are more influential than teachers in helping children be active. | **O** | **O** | **O** | **O** |

*The remainder of the survey will focus on the program(s) you report in Question 20 below.* ***Please consider carefully. You will not have the opportunity to return to this question.***

| 1. Which of the following curricula have any of your sites used **in the previous 7 years (since 2013)**? You might have used all or some of the program components. We expect that you will not be familiar or have used all of these options.    1. Color Me Healthy    2. Food Friends: Get Movin’ with Mighty Moves    3. Food Friends: Fun with New Foods    4. Together, We Inspire Smart Eating (WISE)    5. Grow It, Try It, Like It    6. I am Moving, I am Learning    7. Culture of Wellness    8. Other________________________________ | **YES**  **O** | **NO**  **O** | **Not Sure**  **O** |
| --- | --- | --- | --- |
| *If No to FFMM and FFNF and* if CO was selected as state on landing page:   - Why did you stop using Food Friends? **open comment box*   *If No to WISE* and if AR was selected as state on landing page:   - Why did you stop using WISE? **open comment box* |  |  |  |

| 1. *Based on “yes” answers for curricula in 16 (b, c, or d)…* (BRANCH TO) 2. Please indicate the number of years you used that program. *Give us your best estimate.* 3. What was the last year you offered the program? *Give us your best estimate, including the current school year.* 4. What was your role with the program? *Check all that apply.*     - Lead contact with program administrators and/or teachers    - Teacher in classroom    - Assisted teachers (e.g. order/prepare supplies, teaching assistance)    - Parent education    - Provided approval/funding for use of the program    - Other: ______________________ |
| --- |

1. What else would you like to share about nutrition and physical activity at your center? **Open comment box*

**WISE/FOOD FRIENDS AT YOUR CENTER**

*This section of the survey will ask questions about your center’s use of the WISE/Food Friends program.*

*Please think about the year you* ***most recently*** *used the program.*

*********Please think about your center when it was operating normally, before COVID19.********

| 1. **Please indicate if the following occurred among your center’s staff.** | **Yes** | **No** | **Not sure** |
| --- | --- | --- | --- |
| 1. When we last used it, WISE/Food Friends was going along on a smooth and routine basis. | **O** | **O** | **O** |
| 1. We changed WISE/Food Friends curriculum in a major way. | **O** | **O** | **O** |

| 1. **Which of the following did you do in the most recent year you used the WISE/Food Friends program?** | **Yes** | **No** | **Not sure** |
| --- | --- | --- | --- |
| 1. Offered refresher training in WISE/Food Friends to staff. | **O** | **O** | **O** |
| 1. Offered training/orientation in WISE/Food Friends to new staff. | **O** | **O** | **O** |
| 1. Included WISE/Food Friends costs in your budget. | **O** | **O** | **O** |
| 1. Asked staff about the need to buy new materials to support the use of WISE/Food Friends. | **O** | **O** | **O** |
| 1. Requested technical assistance from WISE/Food Friends staff. | **O** | **O** | **O** |
| 1. Had contact with WISE/Food Friends staff. | **O** | **O** | **O** |
| 1. Made a plan for the best way to integrate WISE/Food Friends into your curriculum. | **O** | **O** | **O** |

| 1. ***(If 16 d is yes)* In your most recent year of using WISE, to what extent did your site do the following:** | **Not at All** | **Somewhat** | **Quite a Bit** | **Very Much** |
| --- | --- | --- | --- | --- |
| 1. Completed WISE activities on a weekly basis. | **O** | **O** | **O** | **O** |
| 1. Used the Windy Wise mascot with WISE lessons. | **O** | **O** | **O** | **O** |
| 1. Used the “Whoo tried it ?” chant with WISE lessons. | **O** | **O** | **O** | **O** |
| 1. Teachers tried WISE foods with children. | **O** | **O** | **O** | **O** |
| 1. Teachers conducted WISE lessons in groups of 6 children or less. | **O** | **O** | **O** | **O** |
| 1. Teachers made positive comments about WISE foods to the children. | **O** | **O** | **O** | **O** |
| 1. Teachers involved children in preparing WISE lessons (e.g., stirring, counting, cutting, grating). | **O** | **O** | **O** | **O** |

| 1. ***(If 16 b or c is yes)* In your most recent year of using Food Friends/Mighty Moves to what extent did your site do the following:** | **Not at All** | **Somewhat** | **Quite a Bit** | **Very Much** |
| --- | --- | --- | --- | --- |
| 1. Completed Food Friends /Mighty Moves activities as outlined by the program. | **O** | **O** | **O** | **O** |
| 1. Used the Food Friends puppets and characters with the lessons. | **O** | **O** | **O** | **O** |
| 1. Used the Food Friends theme song with Mighty Moves lessons. | **O** | **O** | **O** | **O** |
| 1. Teachers tried new foods with children. | **O** | **O** | **O** | **O** |
| 1. Teachers led Mighty Moves lessons by moving with children. | **O** | **O** | **O** | **O** |
| 1. Teachers made positive comments about Food Friends foods to the children. | **O** | **O** | **O** | **O** |
| 1. Teachers engaged children in Mighty Moves activities throughout the day. | **O** | **O** | **O** | **O** |

1. This question gives you an opportunity to share your confidence level with us. How confident are you in your answers to the previous question? We are asking because you might be new to your position or feel unsure. *Slider scale to rate confidence.*
   1. What factors are affecting your confidence in your answer? **Open comment box*

| 1. **Please indicate the extent to which you agree with the following statements.** | **Strongly Disagree** | **Somewhat Disagree** | **Somewhat Agree** | **Strongly Agree** |
| --- | --- | --- | --- | --- |
| 1. In general, WISE/Food Friends is effective in creating attitudes that promote health for children. | **O** | **O** | **O** | **O** |
| 1. In general, WISE/Food Friends is more effective at promoting child health than other programs our site has used. | **O** | **O** | **O** | **O** |
| 1. WISE/Food Friends has improved the overall quality of our teaching practices related to health at our site. | **O** | **O** | **O** | **O** |
| 1. WISE/Food Friends will be effective at preventing obesity for children. | **O** | **O** | **O** | **O** |

| 1. **To what extent did WISE/Food Friends do each of the following: *Slider scale from Not at All to Completely*** |
| --- |
| 1. Met your program’s goals (e.g., licensing, quality ratings, school readiness). |
| 1. Brought benefits (e.g., structured curriculum, positive feedback). |
| 1. Brought challenges (e.g., resource constraints, time). |

| 1. **Please indicate the extent to which you agree with the following statements.** | **Strongly Disagree** | **Somewhat Disagree** | **Somewhat Agree** | **Strongly Agree** |
| --- | --- | --- | --- | --- |
| 1. Plans to support WISE/Food Friends were put in writing for internal purposes (e.g., policy, operations manual). | **O** | **O** | **O** | **O** |
| 1. WISE/Food Friends was included in our weekly classroom schedules. | **O** | **O** | **O** | **O** |
| 1. WISE/Food Friends was evaluated internally at our site (e.g., observations, feedback forms). | **O** | **O** | **O** | **O** |
| 1. WISE/Food Friends had internal staff assigned to support it our site. | **O** | **O** | **O** | **O** |
| 1. WISE/Food Friends had an internal champion or advocate at our site. | **O** | **O** | **O** | **O** |
| 1. WISE/Food Friends was integrated into our overall curriculum. | **O** | **O** | **O** | **O** |
| 1. Our program had adequate funding to support WISE/Food Friends. | **O** | **O** | **O** | **O** |

1. What else would you like to share about using WISE/Food Friends at your center? **Open comment box***________FACTORS INFLUENCING YOUR USE OF WISE/FOOD FRIENDS_______**

*This section of the survey will ask questions about what made it easier or harder for your center to use WISE/Food Friends.*

*Please think about the* ***most recent year*** *you used the program.*

*********Please think about your center when it was operating normally, before COVID19.********

| 1. **For each statement, select the number that best indicates the extent to which your program had/ did the following things.** | **To Little or No Extent**  **1** | **2** | **3** | **4** | **5** | **6** | **To a Very Great Extent**  **7** | **Not Sure** |  |
| --- | --- | --- | --- | --- | --- | --- | --- | --- | --- |
| 1. Champions or advocates existed at our site who strongly supported WISE/Food Friends. | **O** | **O** | **O** | **O** | **O** | **O** | **O** | **O** |  |
| 1. WISE/Food Friends had champions or advocates who garnered additional resources (e.g., food, community donations). | **O** | **O** | **O** | **O** | **O** | **O** | **O** | **O** |  |
| 1. WISE/Food Friends had support from outside of the center (e.g., University, Child and Adult Care Food Program, school district, Head Start admin, families). | **O** | **O** | **O** | **O** | **O** | **O** | **O** | **O** |  |
| 1. **For each statement, select the number that best indicates the extent to which your program did the following things.** | **To Little or No Extent**  **1** | **2** | **3** | **4** | **5** | **6** | **To a Very Great Extent**  **7** | **Not Sure** |  |
| 1. Our center implemented policies to help ensure sustained funding for WISE/Food Friends (e.g., food costs, replacement materials). | **O** | **O** | **O** | **O** | **O** | **O** | **O** | **O** |  |
| 1. WISE/Food Friends was funded through a variety of sources. | **O** | **O** | **O** | **O** | **O** | **O** | **O** | **O** |  |
| 1. WISE/Food Friends had sustained funding at your site (e.g., food costs, replacement materials). | **O** | **O** | **O** | **O** | **O** | **O** | **O** | **O** |  |
| 1. **For each statement, select the number that best indicates the extent to which your program did the following things.** | **To Little or No Extent**  **1** | **2** | **3** | **4** | **5** | **6** | **To a Very Great Extent**  **7** | **Not Sure** |  |
| 1. WISE/Food Friends was well-integrated into your center’s routines. | **O** | **O** | **O** | **O** | **O** | **O** | **O** | **O** |  |
| 1. Systems were in place to support the various needs of WISE/Food Friends. | **O** | **O** | **O** | **O** | **O** | **O** | **O** | **O** |  |
| 1. Our center had adequate staff to complete WISE/Food Friends goals. | **O** | **O** | **O** | **O** | **O** | **O** | **O** | **O** |  |
| 1. **For each statement, select the number that best indicates the extent to which your program did the following things.** | **To Little or No Extent**  **1** | **2** | **3** | **4** | **5** | **6** | **To a Very Great Extent**  **7** | **Not Sure** | |
| 1. Our center made changes to WISE/Food Friends as needed. | **O** | **O** | **O** | **O** | **O** | **O** | **O** | **O** | |
| 1. Our center adapted to changes in the environment for WISE/Food Friends (e.g., turnover, leadership change). | **O** | **O** | **O** | **O** | **O** | **O** | **O** | **O** | |
| 1. Our center made decisions about which components of WISE/Food Friends should continue and which should not continue. | **O** | **O** | **O** | **O** | **O** | **O** | **O** | **O** | |

| 1. **For each statement, select the number that best indicates the extent to which your program did the following things.** | **To Little or No Extent**  **1** | **2** | **3** | **4** | **5** | **6** | **To a Very Great Extent**  **7** | **Not Sure** |
| --- | --- | --- | --- | --- | --- | --- | --- | --- |
| 1. Our center promoted WISE/Food Friends in a way that generated interest (e.g., wall displays, parent communications). | **O** | **O** | **O** | **O** | **O** | **O** | **O** | **O** |
| 1. Our center increased community awareness of WISE/Food Friends. | **O** | **O** | **O** | **O** | **O** | **O** | **O** | **O** |
| 1. Our center demonstrated the value of WISE/Food Friends to others outside of the center (e.g., University, Child and Adult Care Food Program, school district, Head Start admin). | **O** | **O** | **O** | **O** | **O** | **O** | **O** | **O** |
| 1. **For each statement, select the number that best indicates the extent to which your program had/did the following things.** | **To Little or No Extent**  **1** | **2** | **3** | **4** | **5** | **6** | **To a Very Great Extent**  **7** | **Not Sure** |
| 1. Our center planned for future resource needs for WISE/Food Friends. | **O** | **O** | **O** | **O** | **O** | **O** | **O** | **O** |
| 1. Our center had a long-term sustainability plan for WISE/Food Friends beyond our initial year of implementation. | **O** | **O** | **O** | **O** | **O** | **O** | **O** | **O** |
| 1. Our center clearly outlined roles and responsibilities for staff to support WISE/Food Friends. | **O** | **O** | **O** | **O** | **O** | **O** | **O** | **O** |

1. What else would you like to share about factors influencing the use and sustainment of WISE/Food Friends at your center? **Open comment box*

**_______ TELL US ABOUT YOUR CENTER_______**

*This section of the survey will ask questions to understand what it is like to work at your center. These questions will ask about the center as a whole, including but not limited to nutrition and physical activity.*

*********Please think about your center when it was operating normally, before COVID19.********

| Please rate the strength of your agreement with the statements below. | **Strongly Disagree** | **Disagree** | **Neither Agree nor Disagree** | **Agree** | **Strongly Agree** | **Don’t Know/Not Applicable** |
| --- | --- | --- | --- | --- | --- | --- |
| 1. ***Staff members in your organization:*** | | | | | | |
| 1. Have a sense of personal responsibility for improving education and child outcomes. | **O** | **O** | **O** | **O** | **O** | **O** |
| 1. Are willing try new things to improve educational practices. | **O** | **O** | **O** | **O** | **O** | **O** |
| 1. Are open to change in processes. | **O** | **O** | **O** | **O** | **O** | **O** |

| Please rate the strength of your agreement with the following statements. | **Strongly Disagree** | **Disagree** | **Neither Agree nor Disagree** | **Agree** | **Strongly Agree** | **Don’t Know/Not Applicable** |
| --- | --- | --- | --- | --- | --- | --- |
| 1. ***Opinion leaders are people in your organization that tend to influence others. Opinion leaders in your organization:*** | | | | | | |
| 1. Believe that the current practices can be improved. | **O** | **O** | **O** | **O** | **O** | **O** |
| 1. Are willing to try new things. | **O** | **O** | **O** | **O** | **O** | **O** |
| 1. Work cooperatively with administration to make appropriate changes. | **O** | **O** | **O** | **O** | **O** | **O** |

| 1. **In general in my organization, when there is agreement that change needs to happen we have the necessary resources in terms of :** | | | | | | |
| --- | --- | --- | --- | --- | --- | --- |
|  | **Strongly Disagree** | **Disagree** | **Neither Agree nor Disagree** | **Agree** | **Strongly Agree** | **Don’t Know/Not Applicable** |
| 1. Budget or finances | **O** | **O** | **O** | **O** | **O** | **O** |
| 1. Training | **O** | **O** | **O** | **O** | **O** | **O** |
| 1. Facilities (i.e., physical space) | **O** | **O** | **O** | **O** | **O** | **O** |
| 1. Staffing | **O** | **O** | **O** | **O** | **O** | **O** |

1. What else would you like to share about what it is like to work at your center? **Open comment box*

**TELL US ABOUT YOURSELF**

**Now we would like to know more about you. This will help us describe who took our survey.**

| 1. What is your gender? | | | | | | | |
| --- | --- | --- | --- | --- | --- | --- | --- |
| o | Female | o | Male | o | Other | o | Prefer Not to Answer |

| 1. What is your race ? | | | | | | | |
| --- | --- | --- | --- | --- | --- | --- | --- |
| o | Black | o | White | o | American Indian or Alaskan Native | o | Native Hawaiian or Other Pacific Islander |
| o | Asian | o | Two or more races | | | o | Other Race: ___________________ |

| 1. Are you of Hispanic or Latino/a origin? | | | | | | | |
| --- | --- | --- | --- | --- | --- | --- | --- |
| o | Yes | o | No |  |  |  |  |

| 1. Which category includes your age? | | | | | | | | | | | |
| --- | --- | --- | --- | --- | --- | --- | --- | --- | --- | --- | --- |
| o | 18 - 24 | o | 25 - 34 | o | 35 - 44 | o | 45 - 54 | o | 55 - 64 | o | Above 65 |
